# Metformin attenuates metabolic insulin sensitivity and insulin‐stimulated carbohydrate oxidation after high‐intensity exercise training in adults at risk for metabolic syndrome

**DOI:** 10.1111/dom.70478

**Published:** 2026-01-14

**Authors:** Steven K. Malin, Emily M. Heiston, Daniel J. Battillo, Tristan J. Ragland, Anna Ballantyne, Sue A. Shapses, Ankit M. Shah, James T. Patrie

**Affiliations:** ^1^ Department of Kinesiology and Health Rutgers University New Brunswick New Jersey USA; ^2^ Division of Endocrinology, Metabolism and Nutrition, Department of Medicine Rutgers University New Brunswick New Jersey USA; ^3^ New Jersey Institute for Food, Nutrition and Health Rutgers University New Brunswick New Jersey USA; ^4^ Institute of Translational Medicine and Science Rutgers University New Brunswick New Jersey USA; ^5^ Department of Health, Human Performance, and Recreation Pittsburg State University Pittsburg Kansas USA; ^6^ Department of Education University of Virginia Charlottesville Virginia USA; ^7^ Department of Nutritional Sciences Rutgers University New Brunswick New Jersey USA; ^8^ Division of Biostatistics, Department of Public Health Science University of Virginia Charlottesville Virginia USA

**Keywords:** fat metabolism, insulin sensitivity, obesity, physical activity, type 2 diabetes

## Abstract

**Aims:**

Mixed evidence exists on whether metformin adds to or attenuates the insulin‐sensitising effects of exercise. To date, no studies have tested whether metformin differentially impacts exercise training intensity–mediated insulin sensitivity. We tested the hypothesis that metformin would blunt metabolic insulin sensitivity and carbohydrate oxidation in an intensity‐based manner among adults with metabolic syndrome (MetS) risk.

**Materials and methods:**

In a double‐blind, placebo‐controlled trial, participants were randomised to low‐intensity exercise plus placebo (~55% VO_2_max 5 days/week, LoEx + PL, *n* = 22) or metformin (2000 mg/day, LoEx + Met, *n* = 21) and high‐intensity exercise plus placebo (~85% VO_2_max 5 days/week, HiEx + PL, *n* = 24) or metformin (HiEx + Met, *n* = 24) for 16 weeks. A 120‐min euglycaemic–hyperinsulinemic clamp (40 mU/m^2^/min, 90 mg/dL) was conducted pre‐ and post‐treatment to assess metabolic insulin sensitivity (M‐value/insulin). Fasting and insulin‐stimulated carbohydrate oxidation was also assessed using indirect calorimetry. Adipokines (leptin, high molecular weight (HWM), and total adiponectin) were measured at 0 and 120 min of the clamp. Aerobic fitness (VO_2_max) and body composition (DXA) were also analysed.

**Results:**

HiEx + PL increased metabolic insulin sensitivity (*p* = 0.017) while HiEx + Met, LoEx + PL, or LoEx + Met did not. HiEx + PL also raised insulin‐stimulated carbohydrate oxidation and decreased fat oxidation compared with LoEx + Met (both, *p* = 0.008). Increased metabolic insulin sensitivity related to reductions in fasting glucose (*r* = −0.41, *p* = 0.025), VO_2_max (*r* = 0.55, *p* = 0.002), fasting leptin (*r* = −0.54, *p* = 0.01), and weight loss (*r* = −0.60, *p* < 0.001) after exercise and placebo, but not exercise and metformin.

**Conclusions:**

Metformin attenuated metabolic insulin sensitivity and insulin‐stimulated carbohydrate oxidation after high‐intensity exercise training in adults at risk for MetS.

## 1 INTRODUCTION

Exercise and metformin both independently increase 5‐adenosine monophosphate kinase (AMPK).^1^ AMPK is a key mechanism by which each therapy influences glucose regulation via hepatic glucose output suppression and/or insulin‐stimulated glucose disposal.^2^, ^3^ As a result, many have suggested that combining these therapies would yield greater glycaemic control since two of the major organs regulating blood glucose would be impacted compared with either treatment alone. However, the literature on co‐administration of exercise with metformin on blood glucose is equivocal.^4^

We have shown that metformin blunts exercise training–mediated metabolic insulin sensitivity improvements^2^, ^5^ and the reduction in CVD risk factors (i.e., blood pressure, inflammation) compared with exercise training alone.^6^ Others have also reported that metformin attenuates aerobic or resistance exercise‐induced skeletal muscle mitochondrial complex activity as well as mTOR related pathways in middle‐aged to older adults with excess weight.^7^, ^8^ The exact reason for this impaired cellular process remains an area of intense investigation, but metformin is purported to lower oxidative stress by partially inhibiting complex I in the mitochondria, and we have speculated that metformin might attenuate oxidative stress signals from exercise, which, in turn, could blunt AMPK activation as well as glucose regulation adaptations.^9^ The clinical implication of metformin to blunt exercise adaptation for glycaemic benefit though is unclear, as the combination of metformin and moderate to high‐intensity exercise has not been reported to elevate blood glucose levels.^2^, ^5^, ^10^ Rather, it has been shown that the decline in glucose seen with exercise is less, meaning people do not gain the full glycaemic benefit when exercising and taking metformin. However, not all studies agree; some report that metformin increased carbohydrate utilisation during acute high‐intensity interval exercise in relation to insulin sensitivity as measured by the intravenous glucose tolerance test.^11^ Interestingly, this latter finding parallels others whereby chronic treatment with metformin plus exercise favours fasting and oral glucose tolerance derived measures.^12^, ^13^ Collectively, these studies suggest the possibility that metformin may interact with exercise in an intensity‐based manner, particularly since higher intensities of exercise activate AMPK compared with low‐intensity exercise.^14^ Recently, we reported that metformin blunted insulin‐stimulated capillary perfusion as well as large conduit artery function in adults at risk for metabolic syndrome (MetS) after high‐intensity training.^15^ While low‐intensity exercise with metformin also blunted large conduit artery function compared with low‐intensity exercise with placebo, there was no difference in insulin‐stimulated capillary perfusion. Importantly, we noted that fasting glucose decreased following exercise with placebo compared with exercise plus metformin independent of body fat loss.^15^ This latter point is worth considering as adipose tissue secretes factors known as adipokines (e.g., leptin and adiponectin) that are linked to MetS risk and insulin sensitivity.^16^, ^17^ Yet, whether exercise training at low or high intensity with metformin impairs metabolic insulin sensitivity and/or insulin‐stimulated carbohydrate oxidation is uncertain. Therefore, we tested the hypothesis that metformin would impair exercise training–mediated increases in metabolic insulin sensitivity and insulin‐stimulated carbohydrate oxidation compared with training plus placebo in adults at risk for MetS. We also anticipated that changes in metabolic insulin sensitivity and fuel use would relate to the change in aerobic fitness, glycaemia, and adipokines.

## 2 METHODS

### 2.1 Study design and participants

These are the same participants (age: ~55 years, BMI: ~34 kg/m^2^) that were enrolled in our prior study on metformin and exercise interactions related to vascular insulin sensitivity who had available data for metabolic insulin sensitivity.^15^ Individuals were recruited for this randomised, double‐blind, placebo‐controlled trial and MetS risk was defined according to ATP III criteria as previously done.^18^, ^19^ However, due to recruitment challenges during the COVID pandemic, we used the Framingham Risk Score as an alternative risk factor, given its association with insulin resistance.^20^ Participants were recruited via social media and/or newspaper flyers from the Charlottesville, VA and New Brunswick, NJ communities. Individuals were non‐smoking, weight stable (<2 kg weight change during the last 3 months), free of CVD (e.g., congestive heart failure, cerebrovascular/stroke, etc.), or type 2 diabetes as well as any contraindications to metformin (e.g., renal or liver disease, heart failure, and respiratory disease). Preceding exercise testing, participants underwent a physical exam, including overnight 12‐h fasted blood/urine chemistries and resting electrocardiogram. Participants were excluded from the study if taking medications known to influence insulin sensitivity (e.g., biguanides, GLP‐1 agonists, etc.) or affect vasodilatory function (i.e., α‐blockers). Female participants were asked to indicate status of menses, and none were on oral contraceptives or reported use of hormone replacement treatment. All participants provided written and verbal informed consent as approved by our Institutional Review Board. This study was registered on ClinicalTrials.gov (# NCT03355469).

### 2.2 Randomisation and blinding

Participants were randomised to the four different medication and exercise intensity combinations (LoEx + PL, HiEx + PL, LoEx + Met, HiEx + Met) by way of a sex stratified permuted block randomisation scheme. Per block, eight assignments (i.e., two assignments per medication and exercise intensity combination) were generated in random sequential order. The SAS PLAN procedure was used to generate the randomisation (SAS Institute Inc., Cary, NC). Key research personnel were blinded to participant medication assignment.

### 2.3 Metformin or placebo treatment

In a double‐blind design, pills were distributed via our Clinical Research Center (CRC) pharmacy, and participants were instructed to take metformin or placebo with food.^15^ In short, participants started treatment with 500 mg/day of metformin, and the dose was increased 500 mg/day each week until 2000 mg/day (i.e., 1000 mg twice a day) at week 4. Participants remained at this dose for the remaining 12 weeks. Participants were instructed to return any pills missed to verify compliance, which was defined as ≥70% for all conditions. The last metformin ingestion occurred approximately 24 h prior to post‐testing to minimise any potential acute effects.

### 2.4 Exercise training

Participants were instructed to attend three supervised training sessions (e.g., M, W, and F) using a treadmill and to exercise 2 days a week (e.g., T and Thr) on their own (ambulation). These unsupervised days were half the time of the supervised training sessions and designed to support recovery and promote cardiometabolic health. A maximal oxygen consumption (VO_2_max) test on a treadmill with indirect calorimetry was conducted and heart rate max was identified for monitoring of submaximal exercise intensity.^21^ Low‐intensity exercise was defined as a heart rate corresponding to 55% VO_2_max, whereas high‐intensity exercise was defined as 85% VO_2_max. The amount of time participants exercised varied based on their capacity to expend 400 kcal on supervised days and 200 kcal on unsupervised days. Energy expenditure was matched across groups in order to isolate intensity effects. HR/VO_2_ relationships were established from VO_2_max testing to identify the submaximal training dose. Exercise duration thus varied per person in order to meet energy expenditure goals across the different treatment groups. The first training session was done with indirect calorimetry to confirm these estimations. Submaximal training work rate (e.g., speed and/or incline) was then adjusted throughout training to maintain the designated heart rate from initial VO_2_max testing. Participants' completion of supervised and unsupervised exercise sessions was used to characterise compliance, which was defined as >70% for all treatments. The last exercise session of training was performed in the morning and nearly 24 h prior to clamp testing to minimise any potential acute effects on respective outcomes.

### 2.5 Metabolic control

Participants were requested to refrain from consuming alcohol, caffeine, medications, and engaging in strenuous physical activity 24 h prior to the study visits. A low‐fat American Heart Association based diet consisting of 55% carbohydrates, 15% protein, and 30% fat, with <10% from saturated fat, was provided to standardise diet the day before clamp assessments. Energy requirements were determined via fasted resting metabolic rate (RMR) from indirect calorimetry (Cosmed Quark, Chicago, IL) multiplied by a physical activity factor of 1.2.^21^

### 2.6 Euglycaemic hyperinsulinemic clamp

Participants arrived at the CRC following an approximate 10–12 h overnight fast.^15^ Catheters were placed in each of the antecubital veins for infusion and forearm or dorsal hand veins for blood draws. A primed (250 mU/m^2^/min) constant infusion (40 mU/m^2^/min) of insulin (Humulin R U‐500; Eli Lilly and Company, Indianapolis, IN) diluted in saline consisting of 4% (vol/vol) of the participant's own blood was delivered via peristaltic infusion pumps (Harvard Apparatus, Holliston, MA) for 120 min. This insulin dose was selected because it is anticipated to suppress endogenous glucose production and promote skeletal muscle glucose metabolism as originally proposed by DeFronzo et al.^22^ Prior to insulin infusion, expired air (VO_2_ and VCO_2_) was collected for approximately 15 min using a ventilated hood and indirect calorimetry to determine basal substrate oxidation. Plasma glucose samples were collected and analysed every 5 min to adjust the glucose infusion rate and maintain plasma glucose at 90 mg/dL. Insulin, lactate, and free fatty acids (FFA) were obtained at 0, 90, 105, and 120 min of the clamp. The last 30 min were averaged and used to define the steady‐state period of the clamp. Because skeletal muscle is the primary tissue for glucose disposal during insulin stimulation,^23^ we defined skeletal muscle insulin sensitivity as the glucose metabolised (M‐value) divided by steady‐state plasma insulin. Insulin‐stimulated suppression of FFA was calculated as: [1 − (FFA_clamp_/FFA_fast_) × 100%] as a proxy for adipose insulin sensitivity. Respiratory gases were analysed during the steady‐state while the subject rested in the supine position. This was done to assess insulin‐stimulated fuel use and was defined as substrate use_clamp_ − substrate use_fast_. Non‐oxidative glucose disposal (NOGD) was calculated as: glucose metabolised infusion rate − total carbohydrate oxidation rate. Homeostatic model assessment (HOMA‐IR), a surrogate for hepatic insulin resistance, was also calculated as fasting glucose (mg/dL) × fasting insulin (μU/mL)/405 as we have done before.^24^ The adipokines leptin, total adiponectin, and high molecular weight (HMW) adiponectin were measured at 0 and 120 min of the clamp as biochemical indices of endocrine function of fat tissue.

### 2.7 Biochemical analysis

Plasma glucose was collected in lithium heparinised tubes and measured immediately following collection via the glucose oxidase method (YSI Instruments 2300, Yellow Springs, OH). Insulin, FFA, lactate, total‐ and HMW‐adiponectin, as well as leptin samples were centrifuged at 4°C for 10 min at 3000 rpm and subsequently frozen at −80°C. Insulin and FFAs were collected in EDTA vacutainers with aprotinin and analysed using an ELISA (ALPCO, Salem, NH) and colorimetric assays (FujiFilm Wako, Richmond, VA), respectively. Leptin, total adiponectin, and HMW‐adiponectin were collected in EDTA vacutainers and analysed using ELISAs (R&D Systems, Inc., Minneapolis, MN). Lactate was collected in sodium fluoride vacutainers and analysed via the lactate oxidase method (YSI Instruments 2300, Yellow Springs, OH).

### 2.8 Sample size estimation

The primary outcome was metabolic insulin sensitivity, and we estimated metabolic insulin sensitivity variability from our prior work to obtain the present study sample size estimate.^5^ With 15 subjects per treatment intervention, we expected to have at least an 80% chance of rejecting the null hypothesis that the pre to post‐treatment relative change in insulin sensitivity is the same for any pair of treatment interventions if the true underlying pre‐ to post‐intervention relative change in insulin sensitivity differs on average by more than 52% between two treatments. In computing the minimum detectable effect size, we assumed a priori the underlying distribution for pre and post‐treatment relative change in insulin sensitivity is log normal, irrespective of the treatment intervention. We further assumed the measurement variability in the pre to post‐treatment change in log_10_(insulin sensitivity) is the same regardless of the treatment intervention and does not exceed 0.33 units. To account for a total of six pairwise inter‐treatment comparisons, a Bonferroni corrected experiment‐wise type I error of 0.05 was used as part of the sample size formula input information.

### 2.9 Statistical analysis

The PROC MIXED procedure of SAS version 9.4 (SAS, Institute Inc., Cary, NC) was used to conduct analyses. Due to challenges with data collection primarily during COVID‐19 as well as issues with scheduling, we were unable to complete intent to treat analysis given the large number of participants that needed to be excluded as described before.^15^ Details on study enrolment and dropouts were previously published.^15^ In short, we completed data analyses, as done for the primary outcome, on those participants who had complete pre‐ and post‐intervention data realising that this strategy may lead to “missing‐data” induced estimation biases unless the missingness in the outcome data is completely at random (MCAR).^25^ All primary and secondary outcome data were statistically analysed by way of a *full‐likelihood* linear mixed model (LMM) analysis of covariance (ANCOVA) approach. For the covariate adjusted factorial cell‐means linear contrasts, pairwise comparison‐wise *p*‐values were obtained and then the Benjamini and Hochberg false discovery procedure was applied to the complete set of six comparison‐wise *p*‐values to determine among the set of six comparison‐wise *p*‐value those that would be small enough to meet the threshold for null hypothesis rejection if Benjamini and Hochberg cumulative false discovery rate is set at 0.05. For the covariate adjusted marginal mean contrasts, the contrast is only valid if the null hypothesis for metformin by exercise interaction fails to be rejected at *p* ≤ 0.05 level. Marginal mean linear contrasts were rejected as equalling 0 based on a two‐sided comparison‐wise *α* = 0.05 error rate as done before.^15^ Pearson's correlation was performed in exercise and placebo treatments versus exercise and metformin treatments to discern potential associations in fitness, glycaemia, and inflammatory processes that may mediate changes in metabolic insulin sensitivity or insulin‐stimulated fuel use. Data are mean ± SD, and when statistically significant within effects are observed, confidence intervals (CI) are presented. Please see Data S1 for further details on the statistical analysis plan as well as outcome specific marginal effects and interactions.

## 3 RESULTS

### 3.1 Participants characteristics

The data for age, sex, medication, body composition, and fitness for this study population was published in our previous paper focused on vascular insulin sensitivity. Some of those data are briefly presented here for ease of interpretation.^15^ People were on average middle‐aged (LoEx + PL: 57, HiEx + PL: 54, LoEx + Met: 55, and HiEx + Met: 56 years) and over half of the women in each group were post‐menopausal. Individuals had low fitness (LoEx + PL: 2.1, HiEx + PL: 2.3, LoEx + Met: 2.3, and HiEx + Met: 2.2 L/min) and obesity on average (LoEx + PL: 34, HiEx + PL: 35, LoEx + Met: 33, and HiEx + Met: 35 kg/m^2^).^15^ In fact, our prior work shows that metformin blunted the rise in aerobic fitness (LoEx + PL: 0.13 ± 0.24 L/min, HiEx + PL: 0.15 ± 0.25, LoEx + Met: 0.05 ± 0.25, and HiEx + Met: 0.09 ± 0.24 L/min) independent of changes in body weight and body fat changes (via dual‐energy X‐ray absorptiometry as described before).^15^ While HiEx + PL, LoEx + Met, and HiEx + Met statistically reduced body weight by ~2 kg (LoEx + PL: −1.33 ± 5.57, HiEx + PL: −2.73 ± 3.33, LoEx + Met: −2.60 ± 1.70, and HiEx + Met: −2.14 ± 2.37 kg), only HiEx + PL and HiEx + Met statistically reduced body fat (LoEx + PL: −1.19 ± 2.3, HiEx + PL: −1.29 ± 1.49, LoEx + Met: −0.62 ± 1.77, and HiEx + Met: −0.75 ± 1.12%).^15^

### 3.2 Substrates and insulin

LoEx + PL and HiEx + PL lowered fasting glucose when compared with LoEx + Met and HiEx + Met (marginal effect, *p* < 0.05; Table 1). Regardless, there were no differences within or between treatments for steady‐state glucose levels (Table 1). Similarly, fasting plasma lactate was lower after LoEx + PL and HiEx + PL versus LoEx + Met and HiEx + Met (marginal effect, *p* < 0.05). There was no effect of any treatment on steady‐state plasma lactate levels. Only HiEx + Met statistically increased fasting FFA (within effect, *p* = 0.048, mean change: 0.103 mEq/mL, CI: 0.001, 0.204), though LoEx + PL (*p* = 0.094), HiEx + PL (*p* = 0.093), and LoEx + Met (*p* = 0.119) tended to observe rises. Steady‐state FFA were lower after LoEx + PL (*p* = 0.104), HiEx + PL (*p* = 0.045, mean change: −0.08 mEq/mL, 95% CI: [−0.156, −0.003]), and LoEx + Met (*p* = 0.041, mean change: −0.027 mEq/mL, 95% CI: [−0.054, −0.001]) within treatments, but not HiEx + Met (*p* = 0.477). However, FFA suppression tended to increase with LoEx + PL (within effect, *p* = 0.061) and significantly increased with HiEx + PL (within effect, *p* = 0.008, mean change: 8.00%, 95% CI: [3.03, 12.97]), LoEx + Met (within effect, *p* = 0.004, mean change: 11.27%, 95% CI: [4.11, 18.42]) and HiEx + Met (within effect, *p* = 0.02, mean change: 6.31%, 95% CI: [1.22, 11.41]; Table 1). There was no effect of treatments on fasting insulin, yet HiEx + PL (within effect, *p* = 0.008, mean change: −7.40 μU/mL, 95% CI: [−12.61, −2.20]) and LoEx + Met tended to lower (within effect, *p* = 0.055, mean change: −9.21 μU/mL, 95% CI: [−18.64, 0.23]) steady‐state insulin levels (Table 1).

**TABLE 1:** Substrates and insulin before and after exercise training intensity, with and without metformin.

		LoEx + PL	HiEx + PL	LoEx + Met	HiEx + Met
Fasting					
Glucose (mg/dL)	Pre	91.88 ± 10.29	96.05 ± 10.82	99.87 ± 12.83^a^	97.80 ± 12.15^a^
	Post	90.71 ± 12.52	91.69 ± 8.08	99.76 ± 7.41^a^	98.69 ± 14.90^a^
Lactate (mM)	Pre	0.68 ± 0.21	0.77 ± 0.21^b^	0.93 ± 0.24^a^, ^b^	0.90 ± 0.22^a^, ^b^
	Post	0.76 ± 0.21	0.68 ± 0.32	0.89 ± 0.28^a^	1.03 ± 0.36^a^
FFA (mEq/L)	Pre	0.737 ± 0.206	0.652 ± 0.169	0.702 ± 0.233	0.684 ± 0.160
	Post	0.789 ± 0.173	0.735 ± 0.125	0.771 ± 0.224	0.787 ± 0.189^c^
Insulin (μU/mL)	Pre	17.72 ± 19.26	12.98 ± 10.44	12.57 ± 6.95	11.25 ± 7.19
	Post	14.99 ± 15.32	9.28 ± 5.43	10.06 ± 5.14	10.88 ± 7.34
HOMA‐IR (a.u.)	Pre	4.34 ± 4.90	3.60 ± 2.56	3.41 ± 2.12	2.77 ± 1.82
	Post	3.77 ± 4.57	2.48 ± 1.51	2.58 ± 1.23	2.60 ± 2.31
Steady‐state					
Glucose (mg/dL)	Pre	89.31 ± 4.28	88.88 ± 6.33	88.92 ± 5.01	90.24 ± 4.33
	Post	88.14 ± 4.16	89.77 ± 2.20	91.54 ± 3.77	88.06 ± 4.34
Lactate (mM)	Pre	0.81 ± 0.19	0.86 ± 0.24	1.01 ± 0.23^a^, ^b^	0.89 ± 0.20^a^
	Post	0.87 ± 0.28	0.94 ± 0.36	0.95 ± 0.19	0.96 ± 0.24
FFA (mEq/L)	Pre	0.222 ± 0.159	0.174 ± 0.074	0.249 ± 0.187	0.200 ± 0.087
	Post	0.151 ± 0.085	0.146 ± 0.061^c^	0.169 ± 0.087^c^	0.191 ± 0.110
Insulin (μU/mL)	Pre	87.41 ± 15.04	88.39 ± 20.70	80.05 ± 19.53^a^	78.31 ± 16.53^a^
	Post	81.21 ± 36.85	80.99 ± 16.89^c^	70.84 ± 23.49	73.03 ± 17.83
M‐value (mg/kg/min)	Pre	3.39 ± 2.21	2.80 ± 1.44	2.54 ± 1.05	2.68 ± 1.15
	Post	3.29 ± 2.15	3.54 ± 1.61^c^	2.82 ± 1.41	2.75 ± 1.45
FFA suppression (%)	Pre	66.32 ± 26.09	71.88 ± 11.54	65.24 ± 19.64	72.15 ± 11.44
	Post	80.17 ± 10.89	79.82 ± 8.60^c^	76.60 ± 9.01^c^	78.38 ± 10.65^c^

*Note*: Data are mean ± SD. Fasting glucose (LoEx + PL *n* = 15; HiEx + PL *n* = 16; LoEx + Met *n* = 19; HiEx + Met *n* = 16). Steady‐state (last 30 min average of clamp) glucose (LoEx + PL *n* = 15; HiEx + PL *n* = 17; LoEx + Met *n* = 19; HiEx + Met *n* = 15). Fasting lactate (LoEx + PL *n* = 12; HiEx + PL *n* = 16; LoEx + Met *n* = 18; HiEx + Met *n* = 14). Steady‐state lactate (LoEx + PL *n* = 12; HiEx + PL *n* = 14; LoEx + Met *n* = 17; HiEx + Met *n* = 14). Fasting free fatty acids (FFA, LoEx + PL *n* = 13; HiEx + PL *n* = 16; LoEx + Met *n* = 18; HiEx + Met *n* = 13). Steady‐state FFA (LoEx + PL *n* = 12; HiEx + PL *n* = 17; LoEx + Met *n* = 18; HiEx + Met *n* = 14). Fasting insulin (LoEx + PL *n* = 14; HiEx + PL *n* = 16; LoEx + Met *n* = 20; HiEx + Met *n* = 16). Steady‐state insulin (LoEx + PL *n* = 14; HiEx + PL *n* = 16; LoEx + Met *n* = 19; HiEx + Met *n* = 15). Homeostatic model assessment insulin resistance (HOMA‐IR, LoEx + PL *n* = 14; HiEx + PL *n* = 16; LoEx + Met *n* = 19; HiEx + Met *n* = 16). Glucose metabolised (M‐value, LoEx + PL *n* = 15; HiEx + PL *n* = 17; LoEx + Met *n* = 19; HiEx + Met *n* = 15). FFA suppression (LoEx + PL *n* = 12; HiEx + PL *n* = 16; LoEx + Met *n* = 17; HiEx + Met *n* = 12).

^a^ Significant marginal effect of drug (i.e., exercise plus placebo vs. exercise plus metformin), *p* < 0.05.

^b^ Significant compared with LoEx + PL, *p* < 0.05.

^c^ Significant within treatment effect, *p* < 0.05.

### 3.3 Insulin sensitivity and substrate oxidation

**FIGURE 1:**
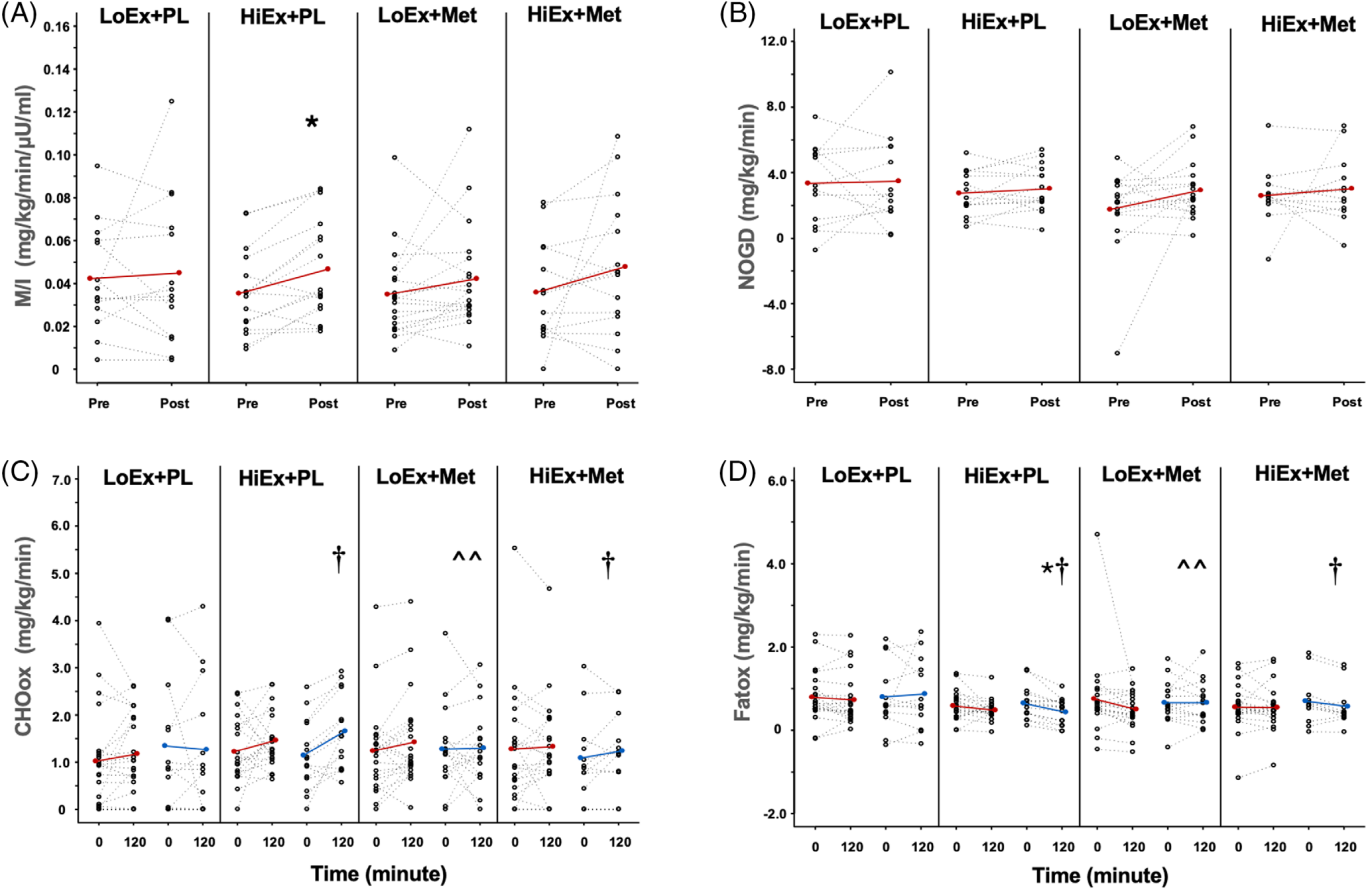
Metabolic insulin sensitivity (A), non‐oxidative glucose disposal (B), and carbohydrate (C, CHOox) as well as fat oxidation (D, Fatox). Data are mean (bars) and individual responses. Metabolic insulin sensitivity (M/I, LoEx + PL *n* = 14; HiEx + PL *n* = 16; LoEx + Met *n* = 19; HiEx + Met *n* = 15). Non‐oxidative glucose disposal (NOGD, LoEx + PL *n* = 13; HiEx + PL *n* = 15; LoEx + Met *n* = 16; HiEx + Met *n* = 12). Carbohydrate oxidation (CHO ox, LoEx + PL *n* = 13; HiEx + PL *n* = 15; LoEx + Met *n* = 16; HiEx + Met *n* = 12). Fat oxidation (Fat ox, LoEx + PL *n* = 13; HiEx + PL *n* = 15; LoEx + Met *n* = 16; HiEx + Met *n* = 12). Significant within treatment effect, *p < 0.05. Significant compared with HiEx + PL, ^^*p* < 0.05. Significant marginal effect of exercise intensity (i.e., high vs. low), †*p* < 0.05.

HiEx + PL raised skeletal muscle metabolic insulin sensitivity (within effect, *p* = 0.017, mean change 0.011 mg/kg/min/μU/mL, 95% CI: [0.005, 0.017]), while HiEx + Met did not (within effect, *p* = 0.747; Figure 1). Similarly, the M‐value was improved only after HiEx + PL (within effect, *p* = 0.017, mean change: 0.74 mg/kg/min, 95% CI: [0.21, 1.28]; Table 1). There was no effect of low‐intensity exercise, with or without metformin, on skeletal muscle insulin sensitivity (Figure 1). HiEx + PL tended to lower HOMA‐IR (within effect, *p* = 0.096) while HiEx + Met did not (within effect, *p* = 0.756; Table 1). NOGD was not impacted by any treatment (Figure 1). In contrast, HiEx + PL raised insulin‐stimulated carbohydrate oxidation (*p* = 0.008) compared with LoEx + Met (Figure 1). However, it is notable that there was a marginal effect for HiEx + PL and HiEx + Met raising for insulin‐stimulated carbohydrate oxidation compared with LoEx + PL and LoEx + Met (*p* = 0.021). There was no effect of treatments on fasting carbohydrate oxidation, although HiEx + PL tended to raise fasting fat oxidation (*p* = 0.068; Table 2). Moreover, HiEx + PL lowered insulin‐stimulated fat oxidation (within effect, *p* = 0.008, mean change: −0.0017 mg/kg/min, 95% CI: [−0.0029, −0.0005]), and this was more than LoEx + Met (*p* = 0.008; Figure 1). There was also a marginal effect for HiEx + PL and HiEx + Met lowering insulin‐stimulated fat oxidation compared with LoEx + PL and LoEx + Met (*p* = 0.01).

**TABLE 2:** Inflammatory responses before and after exercise training intensity, with and without metformin.

		LoEx + PL	HiEx + PL	LoEx + Met	HiEx + Met
Fasting					
Leptin (ng/mL)	Pre	48.77 ± 34.56	41.32 ± 22.10^a^	46.31 ± 27.98	50.45 ± 32.70^a^
	Post	48.43 ± 36.57	29.19 ± 20.64^b^	36.57 ± 25.06^b^	42.76 ± 27.65^b^
Total adiponectin (pg/mL)	Pre	9.87 ± 6.64	8.98 ± 5.33	7.19 ± 4.47	7.36 ± 4.15
	Post	8.50 ± 5.40^b^	8.31 ± 5.03	7.75 ± 6.46	7.07 ± 3.95
HMW adiponectin (pg/mL)	Pre	6.29 ± 5.35	4.48 ± 4.14	3.85 ± 2.85^c^	3.44 ± 1.50^c^
	Post	5.89 ± 4.52	4.05 ± 3.28	3.54 ± 2.32	3.27 ± 1.56
Insulin‐stimulated					
Leptin (ng/mL)	Pre	0.83 ± 13.15	0.94 ± 3.63	2.33 ± 6.82	−0.56 ± 5.49
	Post	−0.08 ± 8.62	0.98 ± 3.79	1.82 ± 4.84	2.36 ± 2.34
Total adiponectin (pg/mL)	Pre	−0.74 ± 1.23	−0.64 ± 1.08	−0.34 ± 0.96	−0.17 ± 1.21
	Post	−0.57 ± 2.62	−0.58 ± 1.26	−2.01 ± 3.34^b^	−0.55 ± 0.93
HMW adiponectin (pg/mL)	Pre	−0.48 ± 1.36	−0.36 ± 0.82	0.08 ± 1.48	−0.06 ± 0.47
	Post	−0.39 ± 0.75	−0.19 ± 0.92	0.31 ± 1.67	−0.08 ± 0.86

*Note*: Data are mean ± SD. Fasting and insulin‐stimulated (i.e., 120–0 min) leptin (LoEx + PL *n* = 10; HiEx + PL *n* = 11; LoEx + Met *n* = 16; HiEx + Met *n* = 13). Fasting and insulin‐stimulated total adiponectin (LoEx + PL *n* = 12; HiEx + PL *n* = 15; LoEx + Met *n* = 16; HiEx + Met *n* = 14). Fasting and insulin‐stimulated high molecular weight adiponectin (HMW‐adiponectin, LoEx + PL *n* = 11; HiEx + PL *n* = 13; LoEx + Met *n* = 16; HiEx + Met *n* = 12).

^a^ Significant marginal effect of exercise intensity (i.e., high vs. low), *p* < 0.05.

^b^ Significant within treatment effect, *p* < 0.05.

^c^ Significant marginal effect of drug (i.e., exercise plus placebo vs. exercise plus metformin), *p* < 0.05.

### 3.4 Adipokines

There was no treatment effect on fasting or clamp‐derived total or HMW‐adiponectin levels (Table 2). However, as with our weight loss results,^15^ leptin was reduced within HiEx + PL (*p* < 0.001, mean change: −11.70 ng/mL, 95% CI: [−16.22, −7.18]), LoEx + Met (*p* = 0.004, mean change: −9.74 ng/mL, 95% CI: [−15.87, −3.61]), and HiEx + Met (*p* = 0.009, mean change: −7.24 ng/mL, 95% CI: [−0.12.39, −2.08]).

### 3.5 Correlations

**FIGURE 2:**
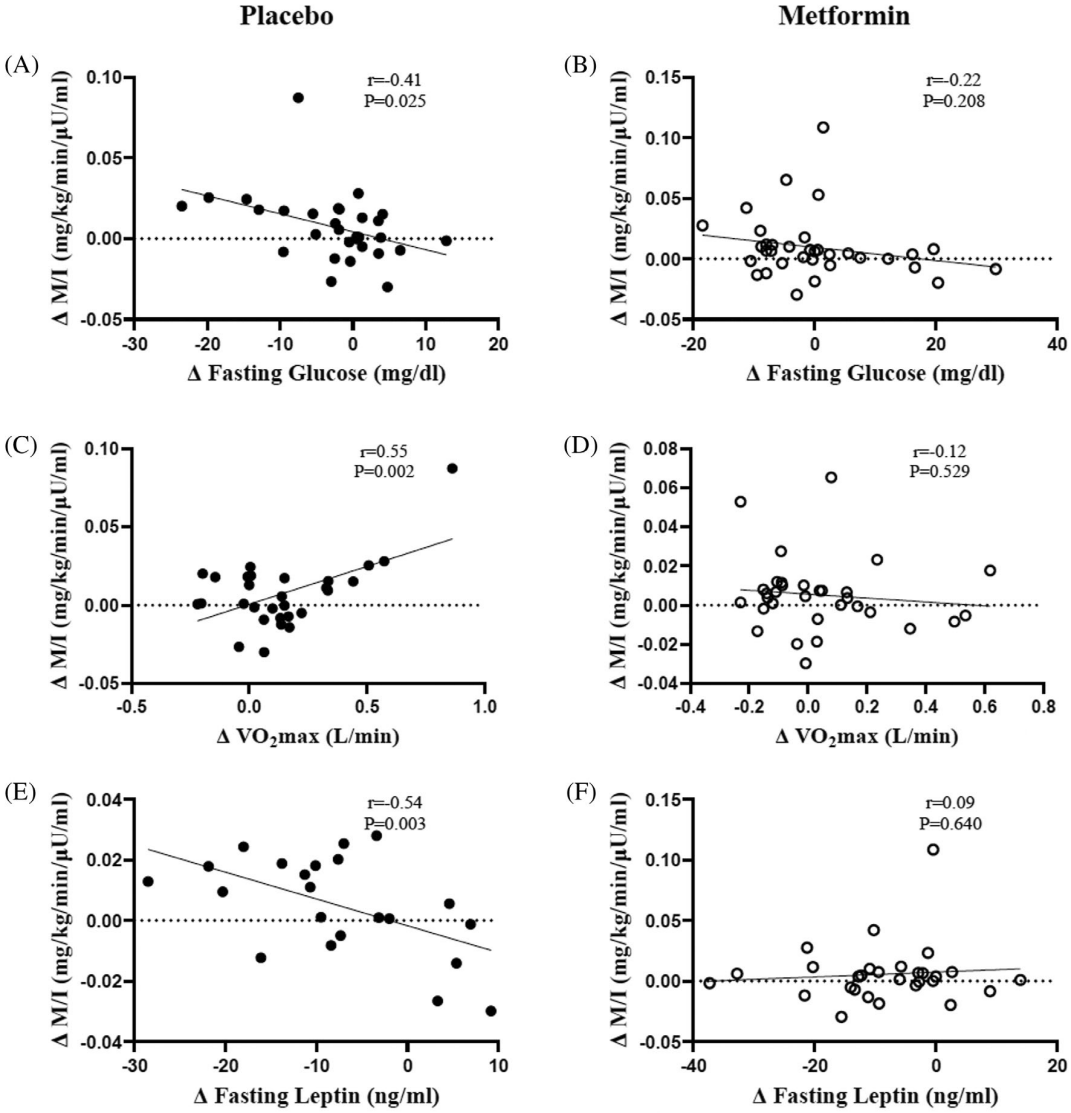
Relationship of metabolic insulin sensitivity with glucose (A, B), VO_2_max (C, D), and leptin (E, F) after exercise with placebo (closed circle) or metformin (open circle).

The increase in metabolic insulin sensitivity seen with exercise and placebo related to reductions in fasting glucose (*r* = −0.41, *p* = 0.025), but this was not observed with exercise plus metformin (*r* = −0.22, *p* = 0.208; Figure 2). An increase in VO_2_max correlated with an increase in metabolic insulin sensitivity following exercise and placebo (*r* = 0.55, *p* = 0.002) but not exercise plus metformin (*r* = −0.12, *p* = 0.529; Figure 2). The increase in metabolic insulin sensitivity related to decreased fasting leptin after exercise and placebo (*r* = −0.54, *p* = 0.01) but not exercise plus metformin treatment (*r* = 0.09, *p* = 0.640; Figure 2). Weight loss was associated with increased metabolic insulin sensitivity after exercise and placebo (*r* = −0.60, *p* < 0.001) but not with exercise plus metformin (*r* = −0.16, *p* = 0.367).

## 4 DISCUSSION

The primary finding of this study was that HiEx + PL statistically increased metabolic insulin sensitivity (i.e., M‐value scaled or unscaled), while HiEx + Met did not. Interestingly, there was no statistical difference in fasting carbohydrate or fat oxidation, yet only HiEx + PL raised insulin‐stimulated carbohydrate oxidation. While this latter finding provides confidence that HiEx + PL uniquely impacted metabolic insulin sensitivity, it is important to recognise this outcome was defined as the M‐value divided by steady‐state insulin levels. We noted that metabolic insulin sensitivity was consistently increased across people following HiEx + PL by approximately 31% when compared with those undergoing HiEx + Met who appeared to raise insulin sensitivity by nearly 33% on average (Figure 1). This seemingly similar result suggests variability may obscure abilities to detect statistical differences after HiEx + Met. Indeed, *n* = 2 participants from those undergoing HiEx + Met appeared to respond in a pronounced favourable way such that they drove the mean change post‐treatment. The remaining participants, however, undergoing HiEx + Met either declined (*n* = 5) or had subtle to no change, confirming large variability in this group. It should be considered too that people randomised to the metformin group had lower insulin levels on average overall than those randomised to placebo, supporting the correction of the M‐value to insulin to ultimately test metabolic insulin sensitivity from the clamp. Yet, when considering the M‐value alone as a reflection of insulin sensitivity (Table 1), it is noteworthy that HiEx + PL increased the M‐value by 26% whereas HiEx + Met only increased the M‐value by only 2%. These findings together suggest that metformin combined with higher intensities of exercise appear to elicit different effects on insulin‐stimulated glucose metabolism when compared with HiEx + PL. A recent consensus statement by the American College of Sports Medicine raised uncertainty about whether high‐intensity exercise elicits greater insulin sensitivity than lower‐intensity exercise for glucose regulation when energy expenditure is matched.^26^ Indeed, we had reported previously that when exercise was work matched, intensity did not differentially impact metabolic insulin sensitivity derived from an oral glucose tolerance test in middle‐aged to older adults with prediabetes.^27^ While the present work was designed to have work matched conditions (i.e., energy expenditure), the limited rise in insulin sensitivity observed after low‐intensity exercise training, independent of metformin, is somewhat surprising. However, these observations are consistent with prior work in aging adults reporting that only high‐intensity exercise improved clamp‐derived metabolic insulin sensitivity.^28^ Thus, the present study findings confirm to some extent prior work by our team that metformin impairs moderate to high‐intensity exercise training effects on clamp‐derived insulin sensitivity when co‐administered in people with impaired glucose tolerance.^5^ This observation is potentially important for not only explaining potential variation between studies given the population difference (MetS risk vs. prediabetes), clamp insulin dose (40 vs. 80 mU/m^2^/min), as well as training program (aerobic + resistance exercise vs. aerobic only),^5^ but also for providing confidence that metformin may impair higher intensity training adaptations. In fact, other studies have also reported that metformin blunts insulin sensitivity as measured by an oral glucose tolerance test in older adults following aerobic exercise training.^7^ These present study findings may also be clinically relevant as we observed increased skeletal muscle insulin sensitivity after exercise training with placebo correlated with decreases in fasting glucose. In contrast, this association was not observed in those exercising with metformin treatment. Additional work here is needed, particularly in the post‐prandial state, to not only confirm such observations, but also elucidate potential mechanisms. Others though suggest metformin and exercise may favour blood glucose reductions or insulin sensitivity compared with exercise alone.^12^, ^29^, ^30^ An important consideration when comparing studies is the timing of metformin and exercise treatment. Specifically, the present study administered metformin and exercise concurrently, as did prior work showing blunted improvements in insulin sensitivity.^2^, ^5^, ^7^ In contrast, others often demonstrate an improvement in glucose regulation outcomes when exercise is added to people habitually taking metformin.^12^, ^29^, ^30^ This is an important clinical consideration for how to best prescribe metformin with exercise and highlights questions around the best order at which metformin and exercise are provided to optimise glycaemic control over time.

Several reasons might explain how metformin interacts with high intensity exercise to alter insulin sensitivity. It is commonly accepted that higher intensities of exercise drive greater increases in VO_2_max than lower intensities of exercise.^31^ This is clinically relevant as high VO_2_max is an important biomarker for reductions in all‐cause mortality.^32^ Metformin has been documented in most,^5^, ^7^, ^13^ but not all^33^ studies, to attenuate increases in aerobic fitness following exercise training in people with obesity, MetS, and/or impaired glucose tolerance. A proposed mechanism for this blunted oxidative capacity relates to partial inhibition of complex I in the mitochondria that impairs cellular respiration.^7^, ^34^ Interestingly, we report that metformin blunted gains in VO_2_max independent of exercise training intensity,^15^ and here we show that improvements in VO_2_max were directly related to increases in clamp‐derived metabolic insulin sensitivity in people who exercised with placebo, but not with metformin. We interpret this to suggest that the ability of exercise to raise fitness may, in part, be related to mitochondrial adaptations that are blunted and influence insulin sensitivity. However, it should be acknowledged that low‐intensity training alone did not lead to statistically significant increases in metabolic insulin sensitivity despite gains in VO_2_max. This raises the possibility that cardiac adaptations contributed to gains in VO_2_max after low‐intensity training group with only subtle metabolic adaptations. Regardless, this altered mitochondrial adaptation with higher intensities of exercise could be a mechanism underlying differential fuel‐oxidation adaptations observed in prior studies.^35^, ^36^ Consistent with this, we show in the present work that the increase in skeletal muscle metabolic insulin sensitivity coincided with increases in insulin‐stimulated carbohydrate oxidation after HiEx + PL, but not in those assigned to metformin. We interpret this as evidence that constraints in fitness, to some extent, parallel limitations in using glucose as an oxidative fuel source during insulin‐stimulation after higher intensities of training with metformin. Although we did not obtain biopsies in an effort to elucidate cellular mechanisms, these findings highlight the need for additional work to investigate the cellular mechanism(s) by which metformin interacts with exercise to improve precision medicine approaches.

Another consideration is that FFAs may inhibit insulin signalling and thus impair insulin sensitivity.^37^ Previously, we showed that metformin raised fasting FFA after exercise training and this was related to blunted clamp‐derived metabolic insulin sensitivity.^5^ Herein, all treatments tended to raise fasting FFAs, although this was only significant after HiEx + Met. Moreover, steady‐state FFAs levels were lower after each treatment except HiEx + Met. However, FFA suppression was improved across all treatments, reflecting improved adipose insulin sensitivity. Thus, the relevance of FFA shifts in fasting and clamp‐derived steady‐state levels is unclear in this study. This may in part be due to the use of a single dose insulin clamp, compared with a two‐stage clamp with lower insulin dose (e.g., 10 and 40 mU/m^2^/min, respectively) that would provide a better opportunity to assess adipose insulin sensitivity. Another possibility though for understanding the shifts in FFA may relate to weight loss. Weight loss was associated with gains in metabolic insulin sensitivity after exercise with placebo, but not with metformin. Prior work shows that weight loss following exercise training lowers the availability of FFA in parallel with improved metabolic insulin sensitivity.^38^ Thus, our findings suggest that metformin interferes with improvements in metabolic insulin sensitivity after high‐intensity training independent of weight loss and shifts in FFAs. However, we acknowledge the possibility that adipose tissue function could be a potential factor contributing to metabolic insulin sensitivity differences when training with metformin versus placebo.

Previously, we have shown that short‐term exercise training, with or without caloric restriction, favours changes in circulating adiponectin and leptin, and changes in these adipokines related to insulin sensitivity in aging adults with obesity/prediabetes.^16^, ^17^ This is clinically relevant as people with obesity and MetS often have low levels of adiponectin and elevated leptin levels that are suggestive of adipose dysfunction and insulin resistance.^39^ In turn, we analysed plasma for HMW‐ and total‐adiponectin as well as leptin in the current work to test potential relations of adipokines with changes in insulin sensitivity and fuel use. Treatments studied in this study had no effects on fasting or clamp‐derived measures of HMW‐ or total adiponectin. This is somewhat surprising as some^16^, ^40^, ^41^ but not all^17^, ^42^ have reported increased HMW or total adiponectin with exercise training. Differences in patient age and/or circulating adiponectin levels may, in part, explain this. Regardless, our work is consistent with prior studies reporting decreased circulating leptin with exercise training following modest weight loss.^16^, ^17^, ^40^ Interestingly, a reduction in fasting leptin after exercise training with placebo in the current work was associated with increased skeletal muscle insulin sensitivity. Importantly, this observation was not observed in those exercising with metformin. This is intriguing given that metformin is noted to promote weight loss,^43^ and people undergoing exercise and metformin treatment lost nearly 2 kg. However, only high‐intensity exercise training, with or without metformin, reduced body fat as previously reported.^15^ This suggests that reductions in body weight/fat are unlikely to explain entirely the shift in plasma leptin. Nonetheless, we observed that weight loss with exercise training alone may influence leptin and relate to skeletal muscle insulin sensitivity, whereas metformin may blunt this observation. Future work should examine this potential crosstalk between skeletal muscle and adipose tissue after exercise training with and without metformin to identify potential targets for rescue.

This study has limitations that could influence our interpretations. We recognise that associations do not equate to causality and that further studies are needed to elucidate the role of metformin attenuating aspects of VO_2_max after exercise training as well as mechanistic underpinnings of blood glucose regulation to provide clinicians more drug‐exercise guidance for patient care. Although we co‐varied our analysis for age and sex, our modest sample size limits definitive evidence for potential interactions with treatment effects. Further, based on our population studied, we are unable to generalise findings to children or older adults as well as people of different racial backgrounds. Moreover, some participants who had been randomised to one of the four treatments at pre‐testing were not able to complete post‐clamp procedures due to time conflict and/or intravenous catheter issues, such that this reduced the total number of participants available for assessment of metabolic insulin sensitivity and/or specific outcomes within the clamp. Blood analysis was also not able to be performed on some outcomes given technical issues (e.g., hemolysed plasma). People performed their last exercise bout approximately 24 h prior to the clamp, and this may have overestimated training‐induced insulin sensitivity. However, this would likely be specific to high‐intensity exercise since low‐intensity training had no statistical effect on metabolic insulin sensitivity. A consideration though with the variability observed with metabolic insulin sensitivity is food intake and non‐exercise physical activity/sedentary behaviour shifts with these treatments. Additional work here is warranted. Lastly, we did not use glucose or lipid stable isotopes to discern roles of glucose disposal versus hepatic glucose production, and lipid turnover. We also did not use lactate isotopes. This is worth consideration as fasting plasma lactate was higher after exercise training with metformin versus placebo in the present study, and this coincided with blunted reductions in fasting glucose after the combined treatment. While this finding is consistent with established roles of lactate as a gluconeogenic precursor, further work here is warranted to determine potential clinical relevance in glycaemic control related mechanisms. It should also be noted that we cannot determine the directionality or causality of increased carbohydrate oxidation and insulin sensitivity. Thus, it remains possible that greater skeletal muscle glucose uptake leads to more carbohydrate utilisation, or enhanced carbohydrate reliance promotes more insulin‐mediated glucose disposal. In either case, the association between greater insulin‐stimulated carbohydrate use and improved glucose control suggests that fuel utilisation may be an important mechanism contributing to glucose homeostasis. Despite these limitations, we are confident our study allows for conclusions on the interaction of metformin with exercise training intensity since we used the gold‐standard euglycaemic clamp technique with indirect calorimetry to assess peripheral or skeletal muscle insulin sensitivity, and an exercise program of sufficient volume.

In conclusion, we show that metformin and high‐intensity exercise training do not increase metabolic insulin sensitivity compared with high‐intensity exercise training alone in adults at risk for metabolic syndrome. A possible explanation for these results is that metformin inhibits insulin‐stimulated carbohydrate utilisation following high‐intensity exercise training. These findings may collectively relate to observations that metformin blunts gains in aerobic fitness and alters relationships among weight loss, leptin, and metabolic insulin sensitivity. Thus, additional work is warranted to understand how to best prescribe metformin and exercise given these are respective first‐line therapies for combating hyperglycaemia. Furthermore, head‐to‐head drug‐exercise studies may be needed as our work and others consistently report that combining two seemingly good therapies does not equate to an additive benefit, and if anything, appears to oppose each other. Long‐term follow‐up is needed as well to identify the clinical consequences of the work.

## AUTHOR CONTRIBUTIONS

S.K.M. conceptualised the study design and hypotheses. S.K.M., E.M.H., D.J.B., T.J.R., A.B., S.A.S., and A.N.S. contributed to recruitment, data collection, and/or data analysis. J.T.P. was responsible for statistical analysis. S.K.M. wrote the manuscript, and all authors provided editorial comments as well as approved the final manuscript.

## FUNDING INFORMATION

Work supported by National Institutes of Health RO1‐HL130296 (SKM).

## CONFLICT OF INTEREST STATEMENT

The authors declare no conflicts of interest.

## Supporting information


**Data S1.** Supporting Information.

**Table S1.** Linear mixed model covariate adjusted *p*‐values for metformin marginal effect, exercise intensity marginal effect, and metformin by exercise intensity interaction.

## Data Availability

S.K.M. is the guarantor of this work and, as such, had full access to all the data in the study and takes responsibility for the integrity of the data and the accuracy of the data analysis. These data have not been made publicly available. However, the corresponding author (S.K.M.) can provide further information on the data upon reasonable request.
