# Supplementary material for: Metformin attenuates metabolic insulin sensitivity and insulin‐stimulated carbohydrate oxidation after high‐intensity exercise training in adults at risk for metabolic syndrome
Source: Diabetes Obes Metab. 2026 Jan 14;28(4):2941–52. doi: 10.1111/dom.70478 (PMC12992159; doi:10.1111/dom.70478)
Supplement: Supplementary file 1 — Data S1. Supporting Information. [file DOM-28-2941-s002.docx]

**SUPPLEMENTAL MATERIALS**

***Statistical Methods***: All primary and secondary outcome data were statistically analyzed by way of a *full-likelihood* linear mixed model (LMM) analysis of covariance (ANCOVA) approach. Drug (Met, PL) and exercise-intensity (LoEx, HiEx) represent the key LMM model fixed-effects along with drug by exercise-intensity interaction. For all outcomes, age, sex, pre-intervention BMI, pre-intervention VO_2_max, and pre-intervention outcome served as the pool of potential co-variates. In order to minimize the chance of residual variance inflation due to non-informative covariates being retained in the final model, a *P*≤0.05 null hypothesis rejection criterion was implemented to determine which co-variate(s) would be retained in the final LMM ANCOVA model. Further, since participants were recruited from two study-sites (i.e., UVA/Rutgers), study-site was considered a LMM ANCOVA model random-effect. Homogeneity of residual variance, an assumption required for traditional linear model ANCOVA hypothesis testing, was relaxed by specifying a heterogenous variance covariance structure in which the estimate for residual variance was allowed to differ among the 4 different metformin and exercise intensity treatment combinations. Within-study arm hypothesis testing was conducted via a non-covariate adjusted LMM linear contrasts of the pre and post-intervention outcome means and within-study linear contrasts were rejected as equaling 0 based on a two-sided comparison-wise α=0.05 error rate. Between-study-arm hypothesis testing was conducted via covariate-adjusted linear contrasts of the LMM ANCOVA cell- means a well as covariate-adjusted linear contrasts of the LMM ANCOVA marginal means (i.e., metformin vs. placebo or high intensity vs. low intensity). For the covariate adjusted factorial cell-means linear contrasts, pairwise comparison-wise *P*-values were obtained and then the Benjamini and Hochberg false discovery procedure was applied to the complete set of 6 comparison-wise *P*-values to determine among the set of 6 comparison-wise *P*-value those that would be small enough to meet the threshold for null hypothesis rejection if Benjamini and Hochberg cumulative false discovery rate is set at 0.05. For the covariate adjusted marginal mean contrasts, the contrast is only valid if the null hypothesis for metformin by exercise interaction fails to be rejected at *P*≤0.05 level. Marginal mean linear contrasts were rejected as equaling 0 based on a two-sided comparison-wise α=0.05 error rate.

For presentation purposes, all *between groups comparison* *P-values* meeting the Benjamini and Hochberg familywise 0.05 false discovery rejection threshold are reported along with covariate adjusted marginal mean linear contrasts, in which the statistical test *P-*value meet the comparison-wise two-sided *P*≤0.05 rejection threshold. Summary data are presented as mean ± SD or individual responses (i.e. figures).
