# Supplementary material for: Metformin attenuates metabolic insulin sensitivity and insulin‐stimulated carbohydrate oxidation after high‐intensity exercise training in adults at risk for metabolic syndrome
Source: Diabetes Obes Metab. 2026 Jan 14;28(4):2941–52. doi: 10.1111/dom.70478 (PMC12992159; doi:10.1111/dom.70478)
Supplement: Supplementary file 2 — Table S1. Linear mixed model covariate adjusted p‐values for metformin marginal effect, exercise intensity marginal effect, and metformin by exercise intensity interaction. [file DOM-28-2941-s001.docx]

***Table S1***. Linear mixed model covariate adjusted *P*-values for metformin marginal effect, exercise intensity marginal effect, and metformin by exercise intensity interaction.

| Outcome | Marginal Metformin  Effect  (*P*-value)† | Margin Exercise  Intensity Effect  (*P*-value)† | Metformin x Exercise Intensity Interaction  (*P*-value) |
| --- | --- | --- | --- |
| *Insulin Sensitivity* |  |  |  |
| M-value | 0.622 | 0.312 | 0.154 |
| M-value divided by insulin | 0.707 | 0.296 | 0.746 |
| HOMA-IR | 0.665 | 0.340 | 0.243 |
| FFA suppression | 0.299 | 0.557 | 0.849 |
| *Substrates and Insulin* |  |  |  |
| Fasting Glucose | 0.007 | 0.400 | 0.735 |
| Steady-State Glucose | 0.385 | 0.279 | 0.006 |
| Fasting Lactate | 0.031 | 0.763 | 0.343 |
| Steady-State Lactate | 0.778 | 0.064 | 0.745 |
| Fasting FFA | 0.580 | 0.878 | 0.440 |
| Steady-State FFA | 0.391 | 0.269 | 0.168 |
| Fasting Insulin | 0.783 | 0.374 | 0.229 |
| Steady-State Insulin | 0.551 | 0.943 | 0.919 |
| *Substrate Metabolism* |  |  |  |
| Fasting CHO oxidation | 0.584 | 0.391 | 0.870 |
| Clamp CHO oxidation | 0.790 | 0.021 | 0.357 |
| Fasting Fat oxidation | 0.533 | 0.238 | 0.974 |
| Clamp Fat oxidation | 0.778 | 0.010 | 0.438 |
| NOGD | 0.847 | 0.832 | 0.981 |
| *Adipokines* |  |  |  |
| Fasting Leptin | 0.739 | 0.149 | 0.006 |
| Clamp Leptin | 0.249 | 0.471 | 0.585 |
| Fasting Total Adiponectin | 0.229 | 0.842 | 0.282 |
| Clamp Total Adiponectin | 0.148 | 0.218 | 0.266 |
| Fasting HMW Adiponectin | 0.206 | 0.167 | 0.411 |
| Clamp HMW Adiponectin | 0.320 | 0.691 | 0.127 |

† *P*-value is only interpretable when the metformin by exercise interaction *P*-value is > 0.05. FFA = free fatty acids. HMW = high molecular weight. CHO = carbohydrate. NOGD = non-oxidative glucose disposal.
